# Shotgun metagenomics reveals the gut microbial diversity and functions in *Vespa mandarinia* (Hymenoptera: Vespidae) at multiple life stages

**DOI:** 10.3389/fmicb.2024.1288051

**Published:** 2024-03-11

**Authors:** Peng-Kai Yin, Huai Xiao, Zhi-Bin Yang, Da-Song Yang, Yin-He Yang

**Affiliations:** ^1^Yunnan Provincial Key Laboratory of Entomological Biopharmaceutical R&D, Dali University, Dali, China; ^2^College of Pharmacy, Dali University, Dali, China

**Keywords:** *Vespa mandarinia*, gut microbiota, metagenomics, WASP, metamorphosis, metabolism

## Abstract

Wasps play important roles as predators and pollinators in the ecosystem. The Jingpo minority residing in Yunnan Province, China, has a traditional practice of using wine infused with mature wasps as a customary remedy for managing rheumatoid arthritis. The larva of the wasp is also a tasteful folk dish that has created a tremendous market. There is a paucity of survival knowledge, which has greatly restricted their potential applications in food and healthcare. Recent research has highlighted the importance of gut microbiota in insect growth. Nevertheless, there is still a lack of understanding regarding the composition, changes, and functions of the gut microbiota in *Vespa mandarinia* during development. In this research, the gut microbiota were investigated across three growth stages of *Vespa mandarinia* using a metagenomic technology. The result revealed that there are significant variations in the proportion of main gut microbes during the metamorphosis of *Vespa mandarinia*. Tenericutes were found to dominate during the larval stage, while Proteobacteria emerged as the dominant group post-pupation. Through a comprehensive analysis of the gut microbiota metagenome, this study revealed functional differences in the wasp gut microbiota at various growth stages. During the larval stage, the gut microbiota plays a central role in promoting metabolism. Following pupation, the gut microbiota exhibited diversified functions, likely due to the complex environments and diverse food sources encountered after metamorphosis. These functions included amino acid metabolism, compound degradation, and defense mechanisms. This research provides an extensive dataset on the gut microbiota during the metamorphosis of *Vespa mandarinia*, contributing to a deeper understanding of the influence of gut microbiota on wasp growth. Furthermore, this study uncovers a unique microbial treasure within insect guts, which is important for advancing the application of wasps in the fields of food and medicine.

## 1 Introduction

Insects are the most abundant and diverse animals, distributed in almost all niches on the earth (Stork, 2007). Many of these insects have exhibited remarkably medicinal properties or economic importance (Dimarcq and Hunneyball, 2003). Hence, insects have become an indispensable part of human life. *Vespa mandarinia* (*V. mandarinia*), a member of the Hymenoptera Vespidae, is a special entomological resource of Yunnan province, China (Zhou et al., 2019). As a folk medicine of the Jingpo minority, the wine made by *V. mandarinia* has been used to treat rheumatoid arthritis for centuries. The living *V. mandarinia* is soaked in an alcohol product named ‘Baijiu' that contains the toxin of wasp, along with a variety of bioactive components from its body. This empirical formula has been listed in the Chinese Pharmacopeia as having an obvious therapeutic effect (Zhou et al., 2019). Moreover, the larva of the wasp is a delicious ethnic food that won the favor of local people (Feng et al., 2018). Wasps have been consumed by people worldwide for a long time and used as the chosen delicacy for celebration in Japan (Dobermann et al., 2017). Recent scientific data indicated that the wasp larva can offer sufficient and balanced essential nutrients due to its low fat, high protein, and carbohydrates (Jeong et al., 2020). The increasing market demand has made the cultivation of *V. mandarinia* become a main source of income for villagers. However, reliable breeding systems are still deficient, and more knowledge about the growth and development of wasps is needed.

As the main place for digestion and absorption, the distinctive environments of the insect digestive tract have attracted the colonization of abundant and various microbes. Some symbiotic bacteria could affect the host's physiology (Wang et al., 2020). Previous research has indicated that the gut microbiota of insects shows diversified symbiotic functions, such as increasing oviposition, shortening larval development, assisting in food digestion, providing essential nutrition, and protecting against pathogens (Engel and Moran, 2013; Jang and Kikuchi, 2020). Some specific antimicrobial symbionts of wasps can even transfer to the brood cells to assist their larvae in fighting against pathogens (Kroiss et al., 2010). The *Wolbachia* of the parasitic wasp can reportedly interfere with programmed cell death for the production of mature oocytes to maintain reproduction (Dedeine et al., 2001). The type and extent of benefits provided by gut microbiota are highly variable across insects and mainly depend on the host species, as the great distinction in ecology, dietary habits, morphology, and lifecycle of the host. A typical example is the differences in gut bacterial communities between adult and larval dung beetles, which possess disparate genes involved in cellulose degradation and nitrogen fixation that are correlated with their distinct diet composition (Shukla et al., 2016). The gut microbiome of adult *V. mandarinia* was first reported in 2019, providing novel insights into how food habits affect the gut microbiota of social insects (Suenami et al., 2019). Despite this, some articles have reported the diversity of gut microbiota in a few species of wasps (Suenami et al., 2019; Cini et al., 2020). Their variation during growth as well as their role in metamorphosis are still unknown.

With the advances in microbial technologies, it has now become clear that the fungi, protists, bacteria, and archaea are the major components of the microorganisms in insect guts (Schmidt and Engel, 2021). For such extremely heterogeneous microbes, it is an impossible task to discover important ones and identify their function with conventional culture-dependent technologies, as the distinctive environments of the digestive tract make many of these bacteria uncultivable with the existing technologies. The advanced culture-independent technologies, including 16S rRNA gene analysis, DNA fingerprinting, and denaturing gradient gel electrophoresis, especially those genome-based “omics” technologies, have broadened our understanding of the communities of gut bacteria (Nayfach and Pollard, 2016; Bobay et al., 2020). Among the technologies, shotgun metagenomics can simultaneously provide an integrated overview of microbial composition as well as their potential functions at the genetic level (Nayfach and Pollard, 2016).

Herein, this research used shotgun metagenomic sequencing to provide hitherto undocumented evidence of the gut microbiota landscape at different growth stages of *V. mandarinia*. Relevant gene sequencing results revealed that the relative abundance of gut microbiota has changed significantly during the metamorphosis of *V. mandarinia*, and these symbiotic bacteria play growth-promoting roles in the development of the host.

This research provides a comprehensive landscape of the gut microbiota between adults and larvae of *V. mandarinia* through metagenomic analysis. This knowledge could be helpful for the technical improvement in wasp farming. Moreover, this understanding uncovers a unique treasure that can explore a special microorganism resource in insect guts.

## 2 Materials and methods

### 2.1 Insect collection

**Figure 1:**
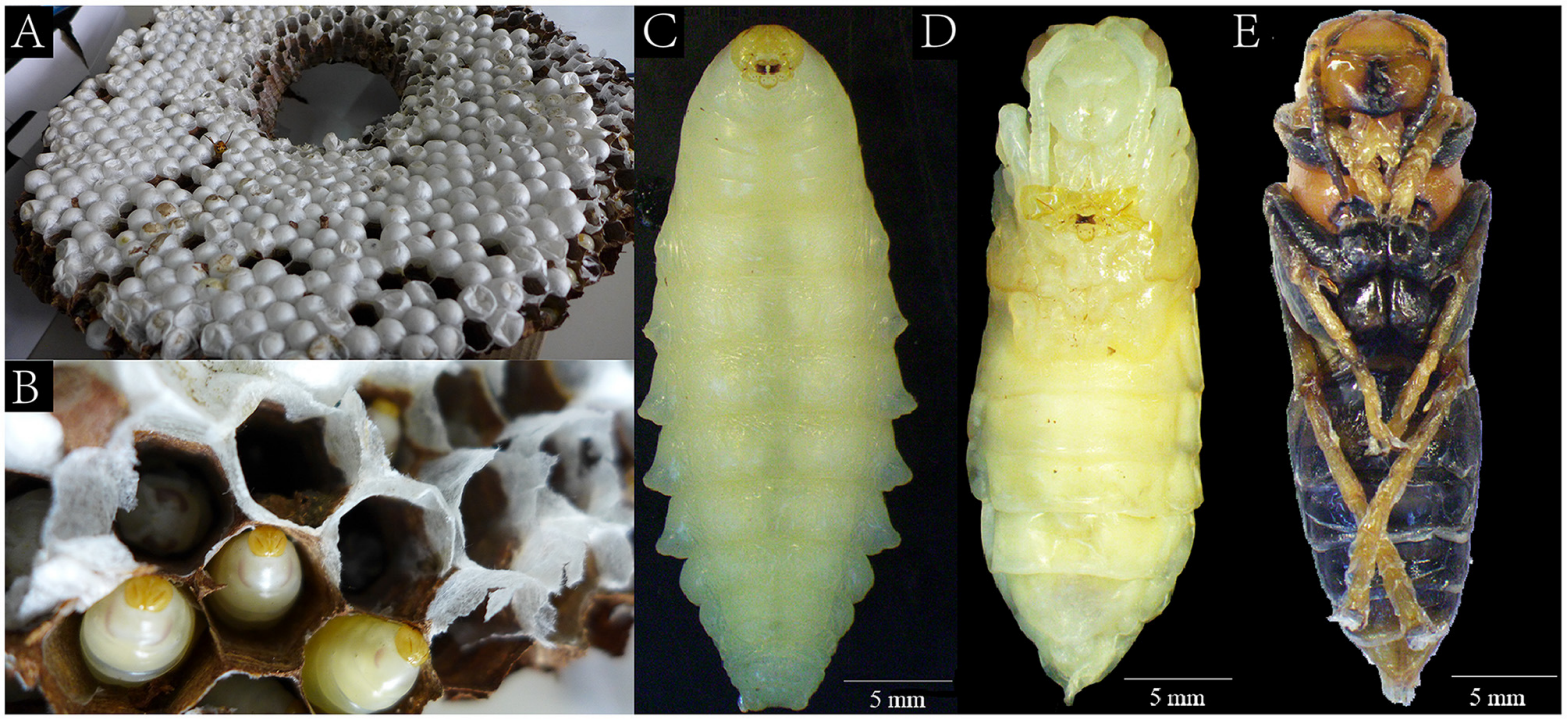
The living environment of the wasps and the wasp morphology in three growth stages. The landscape of transection in wasp hives **(A, B)**. The larva **(C)** of the wasp lived in the hive that was fed by the adult **(E)**, and the nymph **(D)** was developed in the cocoon.

The samples of *V. mandarinia* were collected from Tengchong district, Yunnan Province, China, in September 2020. These samples of *V. mandarinia* were randomly caught in the hive (Figures 1A, B). According to the growth stages, these samples were divided into three groups: larva, nymph, and adult (Figures 1C–E), frozen immediately, and stored in liquid nitrogen.

### 2.2 Dissection and DNA extraction of the *Vespa mandarinia* guts

The samples of *V. mandarinia* in their three distinct forms were randomly selected. Subsequently, 75% alcohol and 0.5% sodium hypochlorite were sequentially used to sterilize the body surface of *V. mandarinia* for 1 min and then washed twice for 1 min in sterile distilled water. Moreover, the anatomical tools were sterilized by autoclaving at 121°C for 30 min. The samples were disintegrated under an aseptic condition, and the gut portions of larval and adult wasps were transferred, respectively, to sterile 1.5 ml microcentrifuge tubes. The TruSeq DNA sample prep kit was used to extract the total meta-genomic DNA from the entomic gut, and then the extracts were analyzed using 1% agarose gel electrophoresis (Qian et al., 2021). An Illumina PE library was constructed: “Y” sticky joints were ligated, eliminating self-ligated fragments through magnetic bead purification, enriching template libraries via PCR, and generating single-stranded DNA fragments using sodium hydroxide (Guo et al., 2021). Single-stranded DNA sequences are amplified into clusters by bridge PCR.

### 2.3 Original metagenomic sequencing of the *Vespa mandarinia* gut

Three paired-end libraries were constructed according to different growth stages, and the extracted metagenomic DNA was sequenced using the Illumina HiSeq technology (Holm et al., 2019). The raw sequencing data were first cleaned and filtered to improve the quality and reliability of the subsequent analysis. The processing method was conducted as follows: (1) filtering these N basic reads with the tail mass below 20, (2) removing below 50 bp reads, (3) removing the read containing adapter and exceeding 2 N bases, and (4) removing reads that are highly similar to the host. The reference wasp genome's assembly accession on NCBI was GCF 014083535.2 (Miller et al., 2022). BWA software aligned the reads to the host DNA sequence and removed highly similar contaminant reads (Oliva et al., 2021). These high-quality reads were used for splicing assembly and gene prediction.

### 2.4 The gene prediction and construction of non-redundant genes

Based on the principles of interactive De Bruijn graphs, the assembly of reads was executed using MEGAHIT software across varying sequencing depths. By progressively increasing the k-mer size, long contigs with few interruptions and branches are achieved through iterative processes (Huang et al., 2020). Contigs ≥100 bp were selected, and MetaGeneMark software (Version 2.10) was used to predict the open reading frame (Wu et al., 2019). The related results were translated into the amino acid sequence. Due to many microbial genes being repeated in the sample, gene sets were obtained by clustering to remove redundancy and enhance data quality. The variation in the abundance of different genes among samples can reflect their general characteristics and discrepancies. The non-redundant gene catalog was constructed to describe the overall information of the genes in these environments. The predicted genes from all samples were clustered by CD-HIT software (95% identity and 90% coverage), and the longest gene of each class was used to build a non-redundant gene database.

### 2.5 Metagenomic analysis

To clarify the composition of the gut microbiome and their variation during the metamorphosis of *V. mandarinia*, the non-redundant genes were compared using BLAST to the non-redundant protein database of the NCBI as a reference database with a set e-value threshold of 10^−5^ (Gao et al., 2017). The function of the gut microbiome was annotated with the eggNOG and KEGG databases. The corresponding Clusters of Orthologous Groups (COG) of proteins were compared to the eggNOG database using BLAST (Huerta-Cepas et al., 2016). The abundance of each COG was calculated by summing the gene abundances associated with it. The gene sets were compared to the KEGG genes database using BLAST (Kanehisa et al., 2017). The resulting alignments of KEGG were then used for functional annotation using KOBAS 2.0 (Xie et al., 2011). The abundance of each functional category, such as pathway, was calculated by summing the gene abundances associated with it.

## 3 Results

### 3.1 Initial sequencing reads and genome filtering results

The whole guts of samples (larvae, nymph, and adults, three groups of each) were extracted to assess their associated microbial communities. A total of 64.47 Gb of metagenomics raw data was obtained by using the Illumina HiSeq. These gut data included genetic information from both host and microbial communities. After quality assessment and host data filtering, the gut microbial metagenomic datasets contained 1.17 Gb. The assembly and binning of profiles are shown in Supplementary Sheet S1. These filtering data were used to analyze the gut microbial diversity and functions in the host and had been uploaded to the NCBI's SRA database with accession number PRJNA751718.

### 3.2 Identification and community diversity of gut microbiota from *Vespa mandarinia*

**Figure 2:**
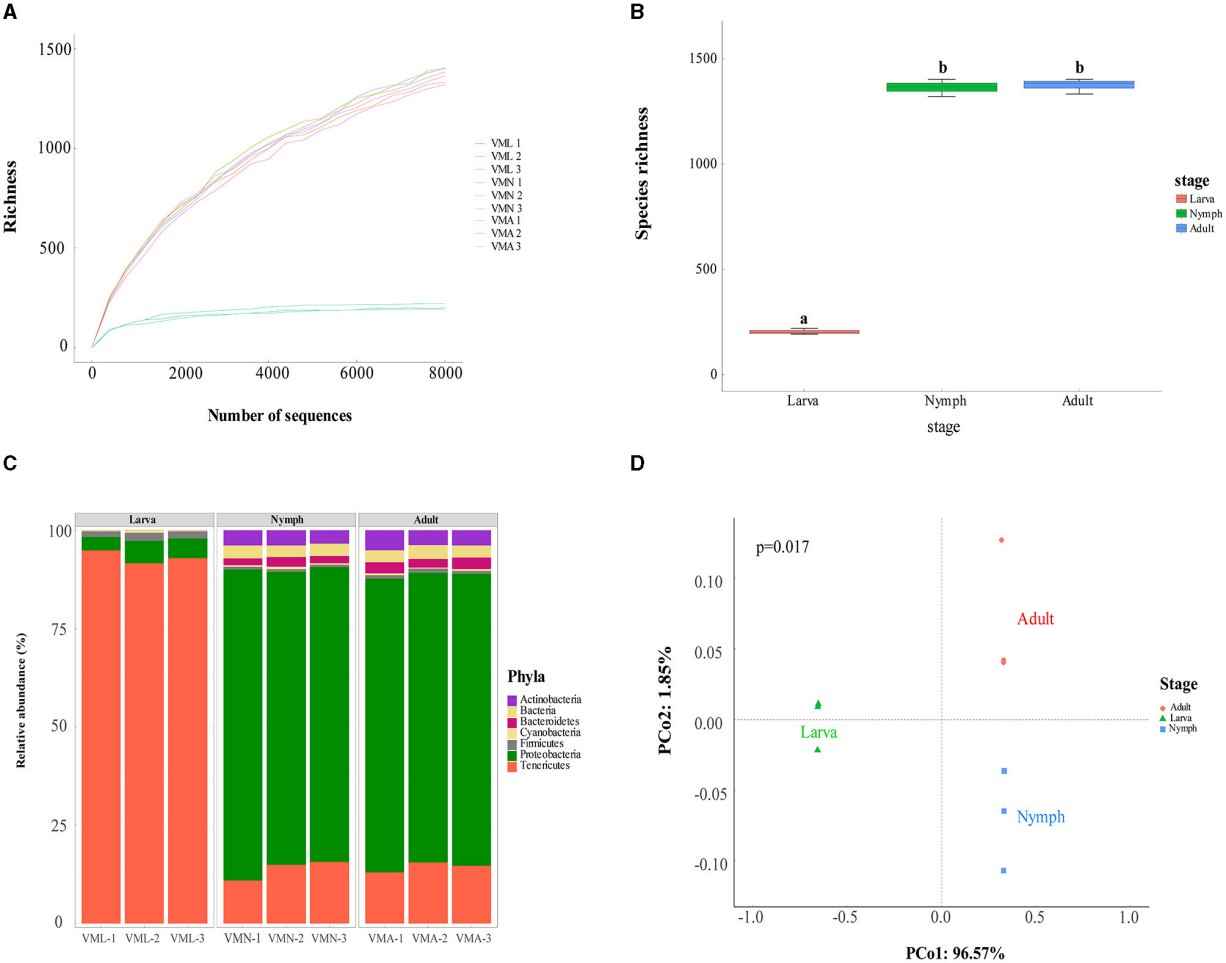
Gut bacterial community dynamics among *Vespa mandarinia*'s three growth stages. VMA represents adult, VMN represents nymph, and VML represents larva. **(A)** The rarefaction curve of species richness, drawn based on the abundance of microbial communities in the samples represented (the sampling gradient was 100 sequences until the maximum depth). These legends were listed in a descending order of richness. **(B)** Boxplot of species richness determined by observed species. Letters above each host species indicate significant differences in the mean values (one-way ANOVA, LSD *post-hoc* test, *p* = 1.31e-08 < 0.01). **(C)** Relative abundance of microbial phyla in different samples. **(D)** PCoA plot based on Bray–Curtis distance for the effect of host development stages on taxonomic diversity (PERMANOVA test with 999 permutations, *p* = 0.017 < 0.05).

Before taxonomic diversity analysis, rarefaction curves of species richness estimates reflected a saturated sampling (Figure 2A). The average Good's coverage was >95%, demonstrating that the sample data were enough for follow-up analyses (Supplementary Sheet S1). The open reading frames (ORFs) were analyzed and predicted from filtered contigs by MetaGeneMark software. On average, the larva had 155 unique ORFs per sample, the nymph had 1,397, and the adult had 1,260 (Supplementary Sheet S1). The taxonomic annotations of those reads were identified using the Non-Redundant protein sequence database (NR database). The species richness of gut bacteria from *V. mandarinia* was compared among different growth stages (Figure 2B). The results exhibited that the gut bacteria were almost the same (one-way ANOVA, LSD *post-hoc* test, *p* = 0.753 > 0.05) in the nymph and adult. However, the gut bacteria in larvae were quite distinctive compared with other stages (one-way ANOVA, LSD *post-hoc* test, *p* = 1.31e-08 < 0.01). The same trend was also observed in the Chao1, Ace richness estimators, Shannon, and Simpson diversity indexes (Supplementary Sheet S1).

The taxonomic analysis revealed that there were seven phyla of microbes distributed in the gut of *V. mandarinia*, which were categorized into 9 classes, 14 orders, 16 families, 19 genera, and 30 species. At the phylum level (Figure 2C and Supplementary Sheet S2), the gut of larvae was occupied mainly by three types of microorganisms, namely, Tenericutes (93.14 ± 1.65%), Proteobacteria (4.76 ± 1.20%), and Firmicutes (1.78 ± 0.25%). However, in nymph and adult groups, the four high-ranking phyla were Proteobacteria (76.23 ± 2.61% in nymph and 74.19 ± 0.61% in adult), Tenericutes (13.84 ± 2.58% and 14.43 ± 1.29%), Actinobacteria (3.69 ± 0.28% and 4.21 ± 0.74%), and Bacteroidetes (1.95 ± 0.37% and 2.60 ± 0.35%). These data indicated that the dominant microbe in the gut of the wasp was rapidly replaced by Proteobacteria after pupation. These changes in the abundance of main gut microbes between different stages of *V. mandarinia* were statistically significant (one-way ANOVA, LSD *post-hoc* test, *p*(Tenericutes) = 3.558e-07 < 0.01, *p*(Proteobacteria) = 0.001052 < 0.01, *p*(Firmicutes) = 7.024e-07 < 0.01). PCoA ordination also suggested that larvae possess a distinct microbiota from other stages. In the scatter plot, the first two principal coordinates, PCO1 and PCO2, explained 96.57% and 1.85% of the data variation, clearly separating each group (Figure 2D).

**Figure 3:**
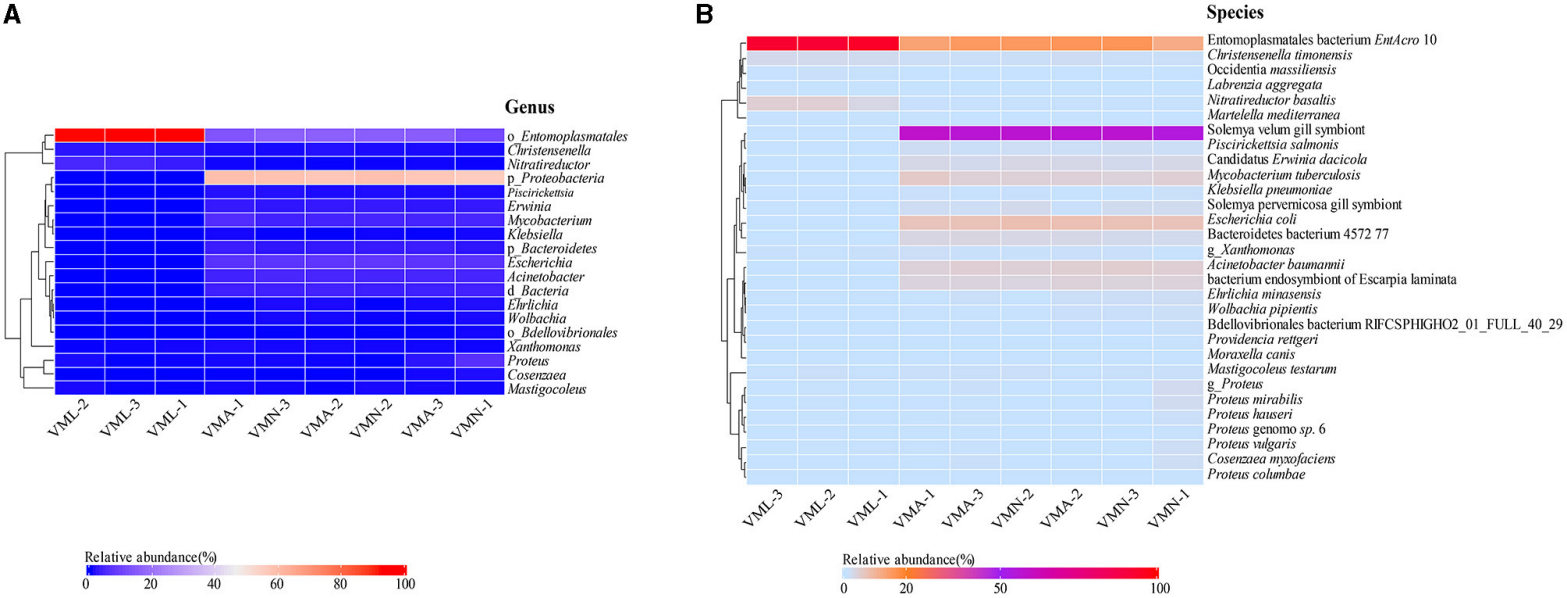
Heatmap of taxa over the *Vespa mandarinia* life cycle at the genera and species level. **(A)** Heatmap showing the relative abundance of genus taxa. **(B)** Heatmap showing the relative abundance of species taxa. Microbial cluster analysis was based on the Spearman distance with the complete-linkage method. The greater the species richness, the darker the color.

A heatmap was prepared using the spearman index to compare the characteristics of the bacterial communities between groups at the genus level (Figure 3A and Supplementary Sheet S3). In the larval groups, the dominant taxa are o_*Entomoplasmatales, Nitratireductor*, and *Christensenella*. The nymph and adult share a similar microbial composition with larvae. The representative bacteria in the nymph and adult wasp mainly belonged to four genera, namely p*_Proteobacteria*, o*_Entomoplasmatales, Escherichia*, and *Mycobacterium*. The representative sequences of operational taxonomic units showed that larvae have different core strains of intestinal bacteria and contain fewer microbial species. A further analysis was conducted on the bacterial community composition among experimental samples at the species level (Figure 3B and Supplementary Sheet S4). The result revealed that the proportion of gut microbial species showed significant heterogeneity across different life stages. Although gene sequences can provide genetic information about microorganisms, they may not be sufficient to determine the species level. The aligned result would provide guidance for the subsequent functional analysis. In the larval group, the main microbe is associated with a bacterium called EntAcro10 and accounts for the highest proportion (Supplementary Sheet S4), which is also symbiotic with ants (Sapountzis et al., 2018), followed by *Nitratireductor basaltis, Christensenella timonensis*. In the adult and nymph groups, the main microbe is associated with *Solemya velum* gill symbiont, which accounts for half of gut microbes, followed by EntAcro10, *Escherichia coli* (Supplementary Sheet S4). The genetic sequences of the two dominant microbial species in the samples and their corresponding results in the NR database are shown in Supplementary Sheet S9.

### 3.3 Functional analysis of gut microbes in *Vespa mandarinia*

Shotgun metagenomic profiles were frequently used to evaluate the functional diversity and potential of microbial communities (Sim et al., 2022). Accordingly, this work analyzed the capabilities of gut microbials from *V. mandarinia* and found their functional divergence with the host growth stage. By searching the KEGG database (Kanehisa et al., 2017), 3,369 genes were aligned from all gut samples, and 2,354 genes could be annotated into 6 categories according to the different functions (Supplementary Sheet S5). Significantly, metabolism was the primary function in larval gut microbes, accounting for 99.85 ± 0.04% of overall annotated sequences. The other functional homologs were present in a very small quantity compared to metabolism. Among adults and nymph groups, metabolism was also enriched, followed by human diseases, environmental information processing, cellular processes, genetic information processing, and organismal systems.

**Figure 4:**
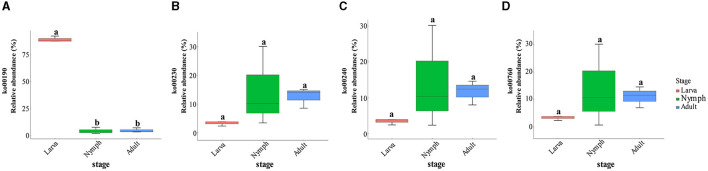
**(A)** Relative abundance and variation of ko00190 (one-way ANOVA, LSD *post-hoc* test, *p* = 2.58e-08 < 0.01). **(B)** Relative abundance and variation of ko00230 (one-way ANOVA, LSD *post-hoc* test, *p* = 0.281 > 0.05). **(C)** Relative abundance and variation of ko00240 (one-way ANOVA, LSD *post-hoc* test, *p* = 0.331 > 0.05). **(D)** Relative abundance and variation of ko00760 (one-way ANOVA, LSD *post-hoc* test, *p* = 0.387 > 0.05).

A further analysis of the 10 most abundant functions at metabolic level 3 was conducted (Supplementary Sheet S6). Oxidative phosphorylation had the highest functional richness in the larva, which was significantly differentiated from other stages (one-way ANOVA, LSD *post-hoc* test, *p* = 1.68e-08 < 0.01). Purine, pyrimidine, nicotinate, and nicotinamide metabolism were enriched in nymphs and adults. Beyond the aforementioned major roles, gut microbes in adult wasps exhibited a greater diversity of functions than larvae. The results of the functional alignments of KEGG pathways and the gene-pathway matching in the samples were presented (Supplementary Sheets S10, S11). For instance, drug metabolism (ko00982), the degradation of chloroalkane and chloroalkene (ko00625), and naphthalene (ko00626). The pathways involved in those main functions were matched on the KEGG database, and their expression in the host was compared (Figures 4A–D). Pathway ko00190 was associated with oxidative phosphorylation, ko00230 was associated with purine metabolism, ko00240 was associated with pyrimidine metabolism, and ko00760 was associated with nicotinate and nicotinamide metabolism.

**Figure 5:**
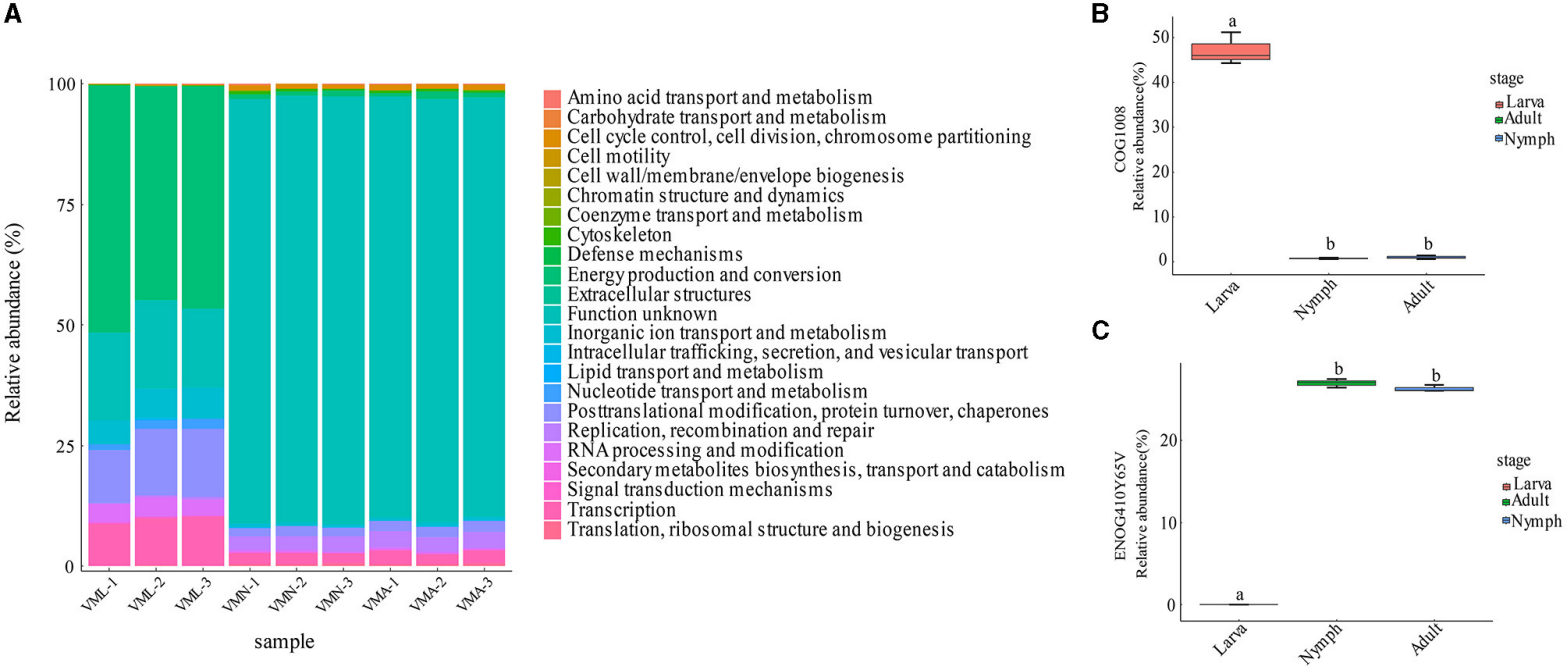
The characteristics of wasp gut microbial homologous genes based on the eggNOG database. **(A)** The functional proteins contained in the wasp gut microbes. **(B)** Relative abundance of the gene COG1008 in the wasp three stages. **(C)** Relative abundance of the gene ENOG410Y65V in the wasp three stages.

The EggNOG database is a hierarchical and phylogenetically annotated orthology resource that has collected numerous protein sequences from various organisms (Huerta-Cepas et al., 2016). Therefore, the metagenomic data from the gut microbiota of *V. mandarinia* were analyzed for functional classification through sequence alignment with the cluster of orthologous groups (COGs). In total, 631 COGs were annotated by function, which were assigned into 23 kinds of COG categories (Figure 5A and Supplementary Sheet S7). The top 10 abundant COGs are listed in Supplementary Sheet S8. Among them, COG1008 was the most abundant protein appearing in the larva and significantly differentiated from other stages (Figure 5B, *p* = 2.3e-07 < 0.01). COG1008 was ubiquinone oxidoreductase subunit 4 of chain M (NADH) and extensively involved in energy production and conversion. The other assigned COGs also contributed to the growth and development of larvae, such as COG0733 and COG5176. ENOG410Y65V was the most expressed in nymphs and adults and different from larval stages (Figure 5C). This COG plays significant functions in the retrotransposon protein. The content of other proteins with similar or related functions was also increased after pupation, including ENOG410XQYK and ENOG410XS82.

To sum up, the abundance and function distribution of gut microbial COGs were altered with host growth. Energy production and conversion were central categories in larval gut microbes. However, the gut microbes in adults and nymphs contained many predicted proteins with unknown functions. In addition to these unknown COGs, the preponderant functions are related to DNA replication, recombination, and repair. Following that is genic transcription and posttranslational modification of proteins.

## 4 Discussion

As an important economic insect in Yunnan Province (China), current cultivation technology of wasp cannot meet the increasing demands of food and medicinal usage. Some gut microorganisms contribute to the host's development by regulating the microenvironment of the gut (Zhang J. et al., 2022). To gain more knowledge about the role that gut microbiota play in the growth of wasps, a metagenomic analysis of the three developmental stages of *V. mandarinia* is conducted in this research. The results substantiated that the composition of gut microflora in *V. mandarinia* exhibits diversity. They are changing with the growth of the wasp to meet the needs of development and help their host adapt to a new survival environment.

In current experiments, the results of metagenomic sequencing indicated that Tenericutes, Proteobacteria, Firmicutes, Cyanobacteria, Actinobacteria, Bacteria, and Bacteroidetes are the main species of gut microbiota in *V. mandarinia*. Plenty of studies have shown that those microorganisms have a significant effect on the survival, behavior, oviposition, longevity, and larval development of host insects (Suenami et al., 2019). Moreover, the unique enzyme from insect gut microbiota can facilitate the breakdown of diversified food resources, and those versatile antimicrobial molecules produced by gut microbiota can help their host survive in harsh environments (Poulsen et al., 2011; Jaffar et al., 2022). The discovery of those promising leads with amazing structural architecture and various bioactivities from intestinal microbes have attracted great interest from chemical and pharmaceutical scientists (Um et al., 2016). As a consequence, in the context of the search for new antibiotics and therapeutic strategies from unique microbes in peculiar environments (Engel and Moran, 2013), this work reveals a previously unexplored isolation source from the insect gut.

Research has demonstrated that the composition of the gut microbiota is closely related to the food source of the host insect and their lifestyle (Xia et al., 2020). The gut microbiota communities of insects widely vary according to ecology. *V. mandarinia* is a social insect, effected by the natural ecological environment, and they can consume a wide variety of food types, such as plants and insects. Therefore, adult wasps are generally considered to be “feeding generalists.” There is a certain difference between the diets of larvae and adults. Adult wasps feed on nectar and other sugary substances, while larvae primarily consume protein-rich prey, which provides them with essential nutrients.

In this work, metagenomic data indicated that the gut of *V. mandarinia* was occupied mainly by two core microbial communities, and their abundance was changed with its cyclogeny. Tenericutes occupy a dominant percentage of larvae and was replaced by Proteobacteria after pupation. Previous studies have demonstrated that Tenericutes are directly associated with metabolism, and hosts with a high abundance of Tenericutes exhibit increased insulin sensitivity (Yuan et al., 2021). Consequently, their high concentration is helpful for the nutrient intake of larvae, and this phenomenon has been documented in other insects (Montalban et al., 2022). By comparison, the ability to use diversified food is an effective way to increase the survival rate for adult wasps. It is relatively easier for wasps to acquire food from plants than from animals. Reports have indicated that the degradation ability of Proteobacteria usually has a high degree of diversity; they can ferment carbohydrates (Delalibera et al., 2005), leading to the accumulation of extracellular enzymes for digesting food. The enzymes produced by Proteobacteria play important roles in the digestion of starches and hemicellulose. The aforementioned function can enhance the ability of adult wasps to utilize multiple foods. In addition, Proteobacteria can provide nutrients such as amino acids and nitrogen (Pinto-Tomás et al., 2009). Hence, the dominant position of Proteobacteria after pupation can increase the environmental adaptation of wasps (Zhou et al., 2020). The dominant species of larvae and adults of the wasp were associated with EntAcro10 and *Solemya velum* gill symbiont, respectively. EntAcro10 can utilize glycerol from ant host cells and monosaccharides (Sapountzis et al., 2018). *Solemya velum* gill symbiont is a sulfur-oxidizing chemosynthetic bacterium. The symbionts are contained within specialized gill cells, giving them access to electron acceptor oxygen in addition to sulfide and carbon dioxide for carbon fixation, fueling both the host and the symbionts (Russell et al., 2018). To sum up, as a special organ and huge gene resource for the host, the variable species richness of the gut microbiota is an adaptive regulatory mechanism of *V. mandarinia*. This mechanism can balance the food supply and nutritional requirements for development by adjusting the ratio of gut microbes with different metabolic capabilities.

Further KEGG analysis also confirms that metabolism is the predominant function of the gut microbes in *V. mandarinia*. In larvae, oxidative phosphorylation is the most abundant function, followed by nicotinate and nicotinamide metabolism, purine metabolism, and pyrimidine metabolism. The oxidative phosphorylation genes were expressed at a high level during the larval stage of *V. mandarinia*, which has been observed in other insects (Ren et al., 2022). Sufficient ATP was produced through oxidative phosphorylation for energy supply to promote the rapid growth of insect larvae (Bretscher and O'Connor, 2020). Entomological research has revealed that it only takes about 40 days for a wasp egg to grow into an adult wasp. Considering the limited time for growth and development of *V. mandarinia* and the scarcity of food resources, optimal conditions are necessary for the completion of their life cycle seasonally. The rapid and plentiful energy supply is essential for the survival of wasp larvae (Tan et al., 2015). Despite energy metabolism is still a core mission of the gut microbes after metamorphosis. Other diverse functions of intestinal microbials from adult wasp are also worth attention, especially the metabolic capacity of different substances, including drug metabolism, chloroalkane and chloroalkene degradation, and naphthalene degradation. The degradability of chloroalkene and naphthalene, which are also found in some insects, would enhance insect defense capacity (Zhang Q. et al., 2022). In Supplementary Sheet S6, we observed an increasing trend in the degradation abundance of chloroalkane, chloroalkene, and naphthalene with the lifecycle. Based on this observation, these functionalities would be acquired by wasp adults when foraging in a certain ecological environment. These capacities may generate unique secondary metabolites and help the adult wasp deal with complicated challenges from various environments.

The eggNOG analysis indicated that larval gut bacteria had abundant COG1008 gene, which was associated with the NADH respiratory complex and oxidative phosphorylation to produce ATP (Getz et al., 2018). Whereafter, with the downregulation of oxidative phosphorylation, retrotransposon proteins were significantly increased in the nymph and adult groups. The high expression of retrotransposons proteins has also been found in other wasps (Ye et al., 2022). The transposable elements may inevitably lead to genomic instability, including breaks and gene disruptions, causing cell death (Ayarpadikannan and Kim, 2014). The high expression and expansion of retranscription proteins in wasp would promote transposable element silencing and reduce cellular damage (Ye et al., 2022).

Retrotransposons and viruses are two major types of genetic elements; even some viruses show a common evolutionary origin with retrotransposons (Koonin and Krupovic, 2017). The symbiotic relationships between wasps and viruses have ancient origins, and some species have mutualistic viral relationships with viruses (Roossinck, 2011). Some wasps have formed phylogenetic symbioses with specific viruses, as revealed by viral metagenomes (Leigh et al., 2018). The typical representative of it is the wasp *Diadromus pulchellus*, which carries a virus that delays the replication of other viruses to protect the host (Sylvaine et al., 2005). Although the burst of retrotransposons has been found in many wasps, their reasons and roles are still not entirely revealed. This research found the high expression of retrotransposon proteins in gut microbes from *V. mandarinia* after pupation, providing new direction for further research.

## 5 Conclusion

This work enriched our knowledge about the gut microbiota during the multiple stages of *V. mandarinia*. Based on these data, three aspects of knowledge can be observed: First, the gut microbiota of *V. mandarinia* has a diverse composition; second, the abundance of gut microbes has changed significantly during metamorphosis; and third, the variation of gut microbes in different developmental stages represents an adaptive selection of *V. mandarinia* to utilize their specific functions for survival. Based on these findings, this work reveals the impact of gut microbiota on the survival capacity of *V. mandarinia*. *V. mandarinia* is a member of the Hymenoptera Vespidae. This work has the potential to contribute to the optimization of wasp cultivation techniques in order to meet food and health market demand. Additionally, this study sheds light on the symbiotic relationship between gut microbes and host and uncovers fewer microorganism resources from insect gut.

## Data availability statement

The datasets presented in this study can be found in online repositories. The names of the repository/repositories and accession number(s) can be found at: https://www.ncbi.nlm.nih.gov/, PRJNA751718.

## Author contributions

P-KY: Data curation, Formal analysis, Writing – original draft, Writing – review & editing. HX: Formal analysis, Writing – review & editing. Z-BY: Resources, Writing – review & editing. D-SY: Conceptualization, Funding acquisition, Project administration, Writing – review & editing. Y-HY: Conceptualization, Supervision, Writing – review & editing.

## Supplementary material

Supplementary Sheet S1 Assembly and binning of metagenomic data of *Vespa mandarinia* gut.

Supplementary Sheet S2 The community and abundance of *Vespa mandarinia* gut microbes at the phylum level.

Supplementary Sheet S3 The community and abundance of *Vespa mandarinia* gut microbes at the genus level.

Supplementary Sheet S4 The community and abundance of *Vespa mandarinia* gut microbes at the species level.

Supplementary Sheet S5 The functional characters in the wasp's different growth stages based on KEGG level 1.

Supplementary Sheet S6 The functional characters in the wasp's different growth stages based on KEGG level 3.

Supplementary Sheet S7 The gut microbial functions of *Vespa mandarinia* based on the eggNOG database.

Supplementary Sheet S8 Those COG genes that ranked in the top ten in terms of abundance at different growth stages.

Supplementary Sheet S9 The alignment results of gene data from the two major microorganisms against the NR database.

Supplementary Sheet S10 The functional definition and functional links of the KEGG pathway in the samples.

Supplementary Sheet S11 The table of functional alignment between KEGG pathways and the genes in the samples.
